# Dual-Cell Polymer–Liquid Crystal Device for Independent Modulation of Light Absorption and Scattering

**DOI:** 10.3390/polym18111405

**Published:** 2026-06-05

**Authors:** Chien-Tsung Hou, Xiang-Dong Mi, Mingqian He, Liang-Chy Chien

**Affiliations:** 1 Advanced Materials and Liquid Crystal Institute, Kent State University, Kent, OH 44242, USA; lchien@kent.edu; 2 Corning Incorporated, Corning, NY 14831, USA; hem@corning.com

**Keywords:** dual-cell liquid crystal, simplified electrode architecture, polymer-stabilized cholesteric texture, dye-doped liquid crystal, smart window, optical sensing

## Abstract

Polymer–liquid crystal (polymer–LC) composites enable electrically tunable optical modulation through the coupling of molecular anisotropy and polymer-induced stabilization. However, most dual-cell LC architectures that independently control absorption and scattering rely on four substrates and multiple independently driven electrode layers, resulting in increased fabrication complexity. In this work, a dual-cell polymer–LC device employing a simplified asymmetric electrode architecture is demonstrated to achieve independent control of absorption and scattering within a three-substrate configuration. The device integrates a dye-doped vertically aligned super-twisted nematic (DDVSTN) cell for absorption-based modulation and a reverse-mode polymer-stabilized cholesteric texture (PSCT) cell for electrically induced scattering. The PSCT layer is driven by interdigitated electrodes on the bottom substrate, while the DDVSTN layer is driven by vertical electric fields, preserving electrical decoupling between the two cells. Four distinct optical states—clear, tinted, private, and tinted-private—are achieved through selective voltage addressing. Spectral measurements confirm stable four-state optical modulation with transmittance varying from approximately 60% in the clear state to about 13% in the tinted-private state. The proposed architecture reduces electrode-layer complexity while maintaining independent optical control, providing a fabrication-efficient platform for smart window systems and polymer–LC photonic devices.

## 1. Introduction

Polymer–liquid crystal (polymer–LC) composites constitute a class of adaptive photonic materials in which electrically responsive molecular anisotropy is combined with structural stabilization. The incorporation of polymer networks introduces anchoring constraints and elastic interactions that modify director stability and establish well-defined electro-optical transitions governed by dielectric torque, elastic restoring forces, chiral twisting tendency, and polymer-induced anchoring effects [1,2]. These synergistic interactions enable controllable optical modulation with improved structural robustness.

Electrically switchable smart windows have attracted considerable interest due to their ability to dynamically regulate solar transmission and improve building energy efficiency [3,4,5,6]. LC devices provide electrically tunable optical modulation with fast response and structural versatility, making them promising candidates for multifunctional glazing technologies [1,2]. In LC-based smart windows, optical regulation is typically achieved either through absorption-based tinting or through electrically induced scattering. However, conventional single-layer configurations cannot independently control transmittance and haze, limiting functional flexibility.

To address this limitation, dual-cell architectures integrating complementary LC mechanisms have been proposed [7,8,9]. In these systems, one LC layer modulates light absorption while the other controls light scattering. Most reported implementations employ two independently driven LC cells requiring four substrates and four electrode layers [7]. Although intermediate substrates can sometimes be merged to reduce structural redundancy, the number of electrode layers generally remain unchanged. As a result, fabrication still requires double-sided electrode processing, which increases manufacturing complexity and alignment tolerance requirements [10,11]. Despite these developments, most reported dual-cell LC devices still rely on multiple independently addressed electrode layers and double-sided electrode processing, and approaches that reduce electrode-layer complexity while maintaining independent optical control remain limited. In large-area device fabrication, the number of electrode layers rather than the number of substrates primarily determines processing difficulty, alignment accumulation, and yield stability.

**Figure 1:**
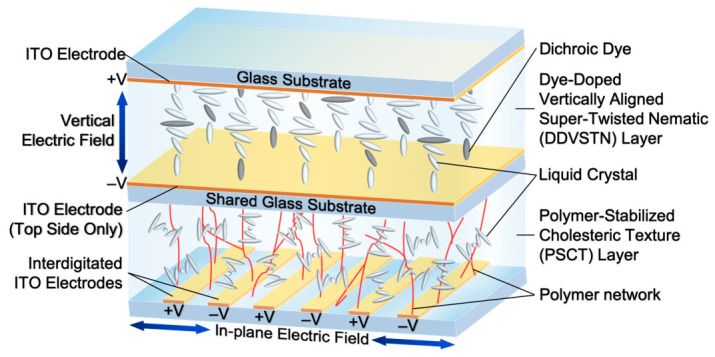
Device architecture of the simplified dual-cell polymer–LC device. The structure consists of a DDVSTN absorption layer and a PSCT scattering layer integrated within a three-substrate configuration. The DDVSTN layer is driven by vertical electric fields between continuous ITO electrodes, while the PSCT layer is driven by in-plane electric fields generated by interdigitated electrodes on the bottom substrate. The middle substrate is shared by the two cells and carries a continuous electrode only for the DDVSTN layer.

To address these challenges, this work demonstrates a dual-cell polymer–LC device employing a simplified asymmetric electrode architecture within a three-substrate configuration. By integrating a dye-doped vertically aligned super-twisted nematic (DDVSTN) layer for absorption-based modulation with a polymer-stabilized cholesteric texture (PSCT) layer for electrically induced scattering, independent control of transmittance and haze is achieved while maintaining electrical decoupling between the two cells. The proposed architecture reduces the number of electrode layers compared with conventional dual-cell designs while preserving independent electro-optical functionality. The device structure of the simplified dual-cell configuration is illustrated in Figure 1. The experimental results demonstrate four distinct optical states—clear, tinted, private, and tinted-private—within the simplified electrode architecture. This approach provides a fabrication-efficient strategy for multifunctional smart window systems and polymer–LC photonic devices.

## 2. Materials and Methods

### 2.1. Device Architecture

A dual-cell LC device was fabricated using a three-substrate, asymmetric electrode configuration to realize independent modulation of absorption and scattering. The bottom cell operated in a reverse-mode PSCT configuration [12,13,14,15,16,17,18,19], while the top cell employed a DDVSTN structure with negative dielectric anisotropy. Both layers were designed to remain optically clear at zero applied voltage.

The asymmetric electrode configuration comprised one continuous indium tin oxide (ITO) electrode on the top substrate and interdigitated ITO electrodes patterned on the bottom substrate, with the middle substrate shared by both cells. The shared middle substrate carried a continuous ITO layer only for the DDVSTN cell, while the PSCT layer was driven exclusively by the interdigitated electrodes on the bottom substrate. Note that this is simpler than and different from previous work using both interdigitated electrodes on one substrate and an ITO electrode on the other substrate [10,20]. This configuration preserved independent electrical addressing of the two LC layers while reducing the number of electrode layers compared with conventional four-electrode architectures.

Although the interdigitated pattern contained two electrically separated regions, they were fabricated from a single ITO layer and were therefore treated as one electrode layer in terms of fabrication complexity. The interdigitated electrodes had a finger width of 10.0 μm and a gap spacing of 10.0 μm. The patterned active interdigitated electrode area was 5 mm × 5 mm, defining the effective in-plane electric-field region for the PSCT layer.

### 2.2. Preparation of the DDVSTN Cell

The DDVSTN cell had a cell gap of approximately 9.3 μm, maintained by glass spacer beads. Vertical alignment was achieved using PI-5661 alignment layer (Nissan Chemical, Tokyo, Japan), which was rubbed twice to generate a preferred tilt direction and facilitate fast recovery in the field-off state, and the two substrates were assembled in an antiparallel rubbing configuration.

The cell was filled with a mixture of 97.02 wt.% negative nematic LC HNG715600-100 (HCCH, Nanjing, China; Δ*n* ≈ 0.153 at 589 nm and 20 °C; Δ*ε* ≈ −12.2 at 1 kHz and 25 °C), 2.0 wt.% dichroic dye M-1012 (Mitsui Fine Chemicals, Tokyo, Japan), and 0.98 wt.% chiral dopant S811 (Merck, Darmstadt, Germany; helical twisting power ≈ 11 μm^−1^). The concentration of the chiral dopant was selected to produce a twist angle (*θ*) of approximately 360°, based on *P* = 1/(HTP·*c*) and *θ* = (*d*/*P*) × 360°, where *P* is the pitch, HTP is the helical twisting power of the chiral dopant, *d* is the cell gap, and *c* is the chiral dopant concentration. The dichroic dye concentration was set to 2.0 wt.% to demonstrate the absorption-based transmittance modulation of the DDVSTN layer. Continuous ITO electrodes on both substrates enabled vertical-field driving.

### 2.3. Preparation of the PSCT Cell

The PSCT cell had a cell gap of approximately 9.3 μm and employed the same vertical alignment layer as the DDVSTN cell. The precursor mixture consisted of 95.27 wt.% positive nematic LC HTG-135200 (HCCH, Nanjing, China; Δ*n* ≈ 0.205 at 633 nm; Δ*ε* ≈ 100 at 1 kHz and 23 °C), 3.99 wt.% reactive mesogenic monomer RM82 (Merck, Darmstadt, Germany), 0.01 wt.% photoinitiator Irgacure 651 (Ciba Specialty Chemicals, Basel, Switzerland), and 0.73 wt.% chiral dopant R811, corresponding to an initial twist angle of approximately 270° prior to polymerization.

The 4 wt.% RM82 concentration was selected to balance vertical-state stabilization and electric-field-driven reorientation. Lower concentrations were insufficient to maintain the vertically aligned state after field removal, whereas higher concentrations resulted in excessive polymer anchoring that suppressed field-induced switching. The selected composition ensured reversible switching between clear and private states.

Polymer stabilization followed established PSCT approaches [12,13,14,15,16]. The filled cell was exposed to 365 nm ultraviolet light at an intensity of 4 mW/cm^2^ for 20 min to polymerize the reactive mesogen without an applied electric field, forming a vertically aligned polymer network that provided localized anchoring and stabilized the LC director configuration [18].

### 2.4. Optical Measurements

Spectral transmittance measurements were performed using a fiber-optic spectrometer (USB4000, Ocean Optics, Orlando, FL, USA) with an unpolarized halogen light source (HL2000-FHSA, Ocean Optics, Orlando, FL, USA) under normal incidence conditions. The applied voltage was supplied at 1 kHz unless otherwise specified.

## 3. Results and Discussion

### 3.1. Reverse-Mode PSCT Operation and Twist-Angle Constraint

In the PSCT layer, the LC behavior under an applied electric field is governed by the competition among dielectric torque, elastic restoring forces, and boundary anchoring [1,2]. While cholesteric LCs intrinsically favor helical ordering, the use of vertical alignment layers imposes a competing constraint that stabilizes a homeotropic (vertically aligned) state in the absence of an applied field. The intrinsic helical pitch (*P*) of a chiral nematic LC is inversely proportional to the concentration of the chiral dopant. In a confined cell, the effective twist angle (*θ*) is determined by the ratio between the cell thickness (*d*) and the pitch, following *θ* = (*d*/*P*) × 360°. Therefore, increasing the dopant concentration reduces the pitch and enhances the intrinsic twisting tendency. In this work, the precursor twist angle is used as a practical parameter to represent this effect [21].

To examine the influence of chiral content on device behavior, precursor twist angles of 90°, 180°, 270°, and 360° were experimentally evaluated. The 90°, 180°, and 270° configurations maintained a stable vertically aligned clear state prior to voltage application and were therefore operable. A qualitative comparison of different precursor twist angles suggests an increasing trend in scattering strength as the twist angle increases from 90° to 270°, with 180° exhibiting stronger scattering than 90°, and 270° providing the most pronounced response. When the twist angle reaches 360°, however, the vertically aligned state can no longer be maintained, and the device becomes inoperable. This behavior indicates that, at 360°, the intrinsic chiral twisting tendency becomes too strong to be balanced by the vertical anchoring, leading to a spontaneous relaxation into a distorted or focal conic texture even in the absence of an applied field, thereby preventing a stable clear initial state.

This enhanced scattering can be attributed to the increased intrinsic twisting tendency, which promotes the formation of more densely distributed distorted domains under an applied electric field. The resulting spatial variations in refractive index leads to stronger light scattering, consistent with previously reported PSCT systems [12,18,19]. These results indicate that the precursor twist angle must be carefully selected to balance two competing effects: sufficient chiral-induced distortion for strong field-driven scattering, and sufficient vertical anchoring to preserve a stable clear state prior to switching. When an in-plane electric field is applied through the interdigitated electrodes, dielectric torque overcomes elastic and anchoring constraints, inducing reorganization of the director field into distorted and focal conic textures (Figure 1, bottom layer). The onset of the transition from the clear state to the private state occurs at approximately 25 V, while 30 V is used to achieve stronger scattering. The threshold voltage depends on dielectric anisotropy, elastic constants, cell gap, and electrode spacing [12,20]. Although direct pitch measurement was not performed, the observed behavior is consistent with the expected relationship between pitch and chiral dopant concentration.

### 3.2. Polymer-Induced Reversibility and Defect Lines

Before polymerization, the precursor mixture can be electrically driven from the vertically aligned configuration into a focal conic texture; however, this transition is generally irreversible in the absence of internal polymer constraints [13]. Once focal conic domains are formed, the system does not reliably recover the initial vertically aligned state after removal of the electric field due to the absence of sufficient internal restoring constraints. Although such a transition is physically possible in the unpolymerized state, it was not intentionally performed prior to polymerization because the initial clear configuration cannot be reproducibly restored.

Polymerization was performed in the vertically aligned state without an applied electric field. During curing, polymer chains grow along the pre-existing director orientation, forming a vertically aligned polymer network distributed throughout the bulk. These polymer structures introduce internal anchoring sites that stabilize the vertically aligned configuration, as widely reported in polymer-stabilized cholesteric systems [18,21].

After polymerization, application of an in-plane electric field generates dielectric torque that drives director reorganization into focal conic and distorted textures, producing the private state. Upon removal of the electric field, dielectric torque vanishes and the vertically aligned polymer network provides a restoring constraint that guides the LC molecules back toward the initial vertically aligned configuration. In this manner, polymer stabilization converts an otherwise irreversible texture transition into a controllable and reversible electro-optical switching process once the applied voltage exceeds the scattering threshold. Reversible switching was qualitatively observed during laboratory operation.

**Figure 2:**
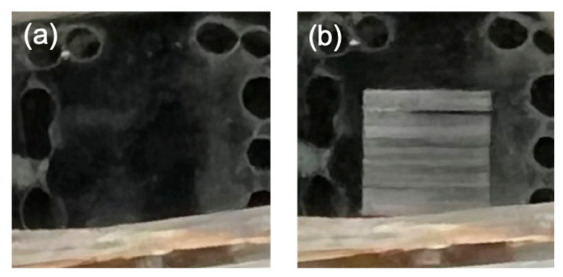
Photographs of the single PSCT cell in the (**a**) clear state and (**b**) private state. Scattering begins at approximately 25 V; 30 V is applied here to produce a stronger scattering performance.

Photographs of the single PSCT cell in the clear and private states are shown in Figure 2a,b, respectively. When the applied voltage exceeds the scattering threshold (25 V), dielectric torque drives director reorganization into focal conic and distorted textures. Slight non-uniform lines parallel to the interdigitated electrode fingers are visible in the private state. These features are attributed to local electric-field distribution and fabrication tolerances associated with electrode patterning. Similar non-uniformities have been reported in interdigitated electrode structures, and optimized electrode geometries may reduce such effects [11]. The electrode geometry in IPS structures plays a critical role in determining the lateral distribution and strength of the in-plane electric field, which is distinct from the role of the cell gap in vertical-field-driven LC configurations. In particular, electrode spacing primarily controls the field penetration and interaction volume, while electrode width and pattern geometry can further influence field uniformity and local distribution. While reducing electrode spacing can enhance local electric-field strength, excessively small feature sizes or non-optimized geometries may introduce field non-uniformity and fabrication-related defects, thereby affecting optical uniformity. Although these parameters were not systematically optimized in the present study, their influence is consistent with previously reported IPS [11,20] and PSCT [15,16,21] systems. A quantitative spectral analysis of the device is presented in Section 3.4 to avoid redundancy and provide a comprehensive evaluation of the integrated system.

### 3.3. DDVSTN Absorption Modulation

The top DDVSTN layer provides absorption-based transmittance modulation. Unlike conventional dye-doped super-twisted nematic (DDSTN) cells, which typically exhibit high absorption in the field-off state that decreases under applied voltage [22,23,24], the vertically aligned DDVSTN configuration is intentionally designed to minimize light absorption at zero voltage. In the absence of an applied field, LC directors and dichroic dye molecules are predominantly aligned perpendicular to the substrates, resulting in minimal absorption and a clear state consistent with the PSCT layer.

Upon application of a vertical electric field, director reorientation increases the effective alignment of dichroic dye molecules relative to the incident light, resulting in enhanced polarization-independent absorption and thereby producing the tinted state (Figure 1, top layer). The applied voltage controls the extent of director reorientation. At low voltages, the optical response changes only slightly, indicating limited reorientation of LC directors. As the voltage increases, more pronounced absorption modulation appears, corresponding to increased alignment of dye molecules with respect to the incident light. At higher voltages (around 25–30 V in the present device), the absorption becomes significantly stronger, leading to a substantial reduction in transmittance. A driving voltage of 30 V was used in this work to clearly demonstrate the tinted state.

This reverse-mode behavior enables independent control of transmittance while preserving a shared clear ground state in the dual-cell architecture. The transmittance decreases continuously with applied voltage, enabling intermediate gray levels between the clear and tinted states. The absorption strength is determined primarily by the concentration of dichroic dye and the effective alignment of the dye molecules relative to the incident light polarization.

In the present device, a dye concentration of 2 wt.% was selected to provide a noticeable transmittance modulation while maintaining a relatively clear initial state. Increasing dye concentration would enhance absorption in the tinted state but may reduce the transmittance in the clear state, resulting in a trade-off between contrast and transparency. The concentration used here was therefore chosen to demonstrate clear modulation behavior rather than to achieve optimal electro-optical performance.

### 3.4. Optical States

**Figure 3:**
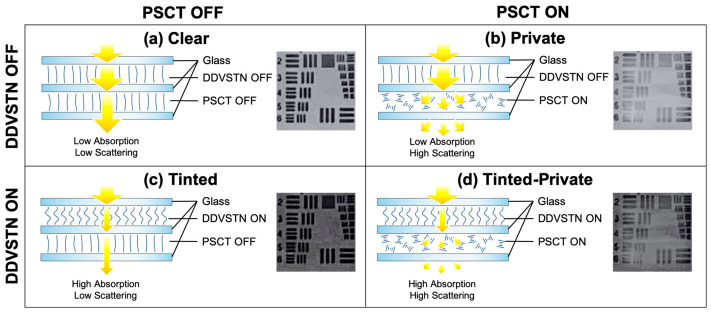
Schematic illustration and corresponding photographs of the four optical states: (**a**) clear, (**b**) private, (**c**) tinted, and (**d**) tinted-private. The photographs were obtained using a USAF 1951 resolution target as the background, with the camera focused on the target to ensure that the image remained sharp in the clear state. The displayed region corresponds to the 5 mm × 5 mm active area of the interdigitated PSCT electrode region. The scattering states primarily exhibit degradation of image clarity and spatial resolution due to electrically induced scattering in the PSCT layer.

The optical response of the simplified dual-cell device is attributed to the independent yet complementary operation of the DDVSTN absorption layer and the PSCT scattering layer. By selectively addressing the two LC cells, four distinct optical states—clear, tinted, private, and tinted-private—can be achieved, as illustrated in Figure 3.

In the clear state (Figure 3a), the background USAF 1951 resolution target remains sharp and clearly distinguishable, indicating low absorption and minimal scattering in the device. In this condition, both LC layers remain in their transparent configurations. The DDVSTN layer is in the field-off vertically aligned state, where the dichroic dye molecules are predominantly oriented along the substrate normal and therefore exhibit minimal effective absorption for normally incident light. At the same time, the PSCT layer remains in the vertically aligned configuration without forming scattering domains. As a result, the device exhibits low absorption and low scattering, corresponding to the highest transmittance among the four states.

When the PSCT layer alone is driven, the device switches to the private state (Figure 3b). In this condition, degradation of image clarity and spatial resolution can be observed through the USAF 1951 target, particularly for higher-spatial-frequency features. The DDVSTN layer remains in the low-absorption configuration, while the PSCT layer transforms into a focal conic or distorted scattering texture under the in-plane electric field. The resulting refractive-index inhomogeneity diffuses transmitted light and increases haze, thereby reducing image clarity through the device.

When a voltage is applied only to the DDVSTN layer, the device enters the tinted state (Figure 3c). In this state, the overall image brightness decreases while the background pattern remains relatively sharp, indicating that the optical modulation is dominated primarily by absorption rather than scattering. This behavior results from field-induced director reorientation in the DDVSTN layer, which increases the effective absorption of the dichroic dye molecules.

When both LC layers are simultaneously activated, the device reaches the tinted-private state (Figure 3d). In this configuration, the DDVSTN layer contributes increased absorption while the PSCT layer simultaneously generates scattering-induced haze. As a result, the device exhibits both reduced transmittance and degradation of image clarity due to the combined effects of absorption and scattering. The present device primarily demonstrates electrically induced degradation of spatial resolution and image clarity through moderate scattering generated by the PSCT layer.

### 3.5. Spectral Performance

**Figure 4:**
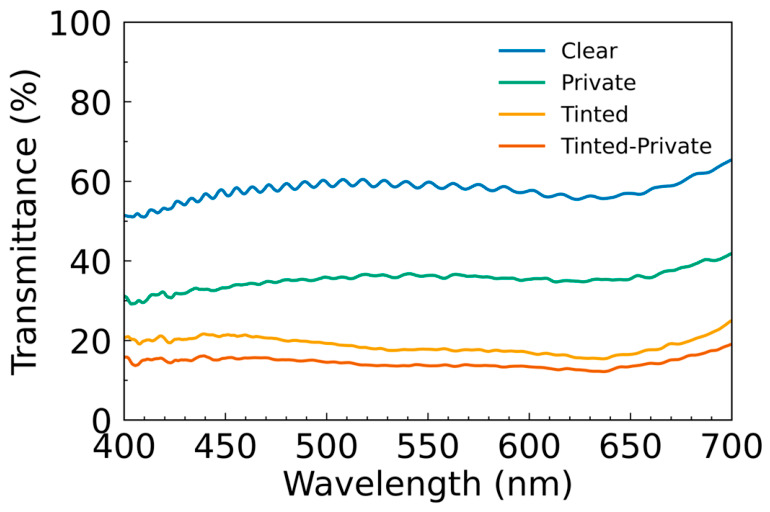
Spectral transmittance of the dual-cell device measured in the clear, private, tinted, and tinted-private states. The measurements confirm the four-state optical modulation resulting from the independent operation of the DDVSTN absorption layer and the PSCT scattering layer.

Figure 4 shows the spectral transmittance corresponding to the four optical states presented in Figure 3. The spectra exhibit four clearly distinguishable transmittance levels without significant overlap or crossing throughout the visible wavelength range, indicating that the four optical states are optically distinguishable.

Within the visible range (400–700 nm), all four spectra remain relatively flat, with the transmittance at 400 nm slightly lower than that at 700 nm. This behavior is attributed to the broadband absorption characteristic of the black dichroic dye, which provides relatively uniform attenuation across the visible spectrum despite the underlying wavelength-dependent absorption outside this range. Although the absorption spectrum of the individual DDVSTN layer was not separately measured, the observed spectral trend is consistent with previously reported characteristics of black dichroic dyes.

Among the four optical states, the clear state exhibits the highest transmittance of approximately 60%. The private state exhibits an intermediate transmittance of approximately 36%, while the tinted state exhibits a lower transmittance of approximately 18% due to increased dye absorption in the DDVSTN layer. The tinted-private state exhibits the lowest transmittance of approximately 13% due to the combined effects of absorption and scattering. These results quantitatively confirm that independent modulation of absorption and scattering is preserved within the simplified electrode configuration.

The optical performance of the present device reflects the trade-offs commonly associated with dual-cell LC architectures. In the clear state, the transmittance is influenced by the combined effects of the dye-doped DDVSTN layer, incomplete suppression of scattering in the PSCT layer, and interface-related optical losses. In the private state, the PSCT layer generates moderate scattering through electrically induced distorted and focal conic textures, resulting in degradation of image clarity and spatial resolution while maintaining partial transmission.

The present device was designed to demonstrate device functionality and independent absorption and scattering modulation within a simplified dual-cell architecture rather than maximize the optical performance of any individual state. Previous studies have demonstrated that stronger scattering and lower off-state transmittance may be achieved through further optimization of material composition, device parameters, and driving conditions [12,13,14,18,19,20,21,24]. Despite the lack of full optimization, the present results confirm the feasibility of independently controlling light absorption and scattering within the simplified dual-cell structure configuration.

### 3.6. Perspective on Optical Sensing Applications

Although originally developed for smart window applications, the polymer–LC composite architecture described here exhibits characteristics that may be relevant for intensity-based optical transduction. It should be noted that no dedicated sensing experiments were performed in this study; the following discussion is intended to outline possible extensions based on the observed electro-optical behavior and established LC physics.

In the PSCT layer, the transition from transparency to scattering occurs at a characteristic voltage threshold determined by the balance among dielectric torque, elastic energy, and surface anchoring [12,21]. Such voltage-dependent optical responses have been widely established in polymer-stabilized cholesteric systems [12,17,18,19]. Previous studies have shown that this threshold is sensitive to variations in material parameters and boundary conditions. Therefore, monitoring changes in switching voltage or scattering intensity could, in principle, provide a mechanism for detecting external perturbations.

In the DDVSTN layer, absorption is governed by the field-dependent orientation of dichroic dye molecules [22,23,24]. Variations in birefringence, effective pitch, or alignment conditions may influence the voltage–transmittance response. Such dependencies suggest that calibrated intensity modulation could potentially be used for sensing applications.

In addition, external stimuli such as mechanical deformation may alter boundary conditions and director configuration in both layers, leading to measurable optical changes. While these effects were not systematically investigated in the present work, they are consistent with previously reported LC-based sensing mechanisms. Similar mechanisms have been reported in LC-based optical sensing systems [25].

Overall, the simplified asymmetric configuration enables a compact platform integrating both scattering and absorption channels. This structure may provide a foundation for future studies exploring multifunctional optical sensing, where systematic experimental validation will be required.

## 4. Conclusions

A simplified dual-cell LC architecture has been demonstrated to achieve independent control of absorption and scattering within a simplified three-substrate configuration. By redistributing the electric field and eliminating one electrode layer, electrical decoupling between the DDVSTN and PSCT cells is preserved while reducing fabrication complexity.

The reverse-mode PSCT design relies on a carefully selected precursor twist angle near 270° and an appropriate RM82 concentration that balances scattering strength, vertical-state stability, and reversible recovery after field removal. Polymer stabilization provides internal restoring constraints that enable repeatable switching following field-induced focal conic formation. By combining the absorption-based DDVSTN layer and the scattering-based PSCT layer, the device supports four optical states—clear, tinted, private, and tinted-private—within a common transparent baseline.

Spectral measurements confirm the four-state operation, with the transmittance varying from approximately 60% in the clear state to about 13% in the tinted-private state, demonstrating a substantial optical modulation range. The demonstrated dual-cell configuration provides a fabrication-efficient pathway toward scalable smart window integration and polymer–LC photonic devices and suggests potential for intensity-based optical sensing applications.

## Data Availability

The data presented in this study are available from the corresponding author upon reasonable request.

## Abbreviations

The following abbreviations are used in this manuscript:

LC	Liquid crystal
PSCT	Polymer-stabilized cholesteric texture
DDVSTN	Dye-doped vertically aligned super-twisted nematic
DDSTN	Dye-doped super-twisted nematic
ITO	Indium tin oxide
